# *trans*-Caryophyllene, a Natural Sesquiterpene, Causes Tracheal Smooth Muscle Relaxation through Blockade of Voltage-Dependent Ca^2+^ Channels

**DOI:** 10.3390/molecules171011965

**Published:** 2012-10-11

**Authors:** Leidiane Pinho-da-Silva, Paula Virgínia Mendes-Maia, Taylena Maria do Nascimento Garcia Teófilo, Roseli Barbosa, Vania Marilande Ceccatto, Andrelina Noronha Coelho-de-Souza, Jader Santos Cruz, José Henrique Leal-Cardoso

**Affiliations:** 1 Laboratório de Eletrofisiologia, Instituto Superior de Ciências Biomédicas, Universidade Estadual do Ceará, Av. Paranjana #1700, Fortaleza 60740-000, Ceará, Brazil; 2 Programa de Pos-Graduação em Fisiologia e Farmacologia, Universidade Federal de Minas Gerais, Avenida Antonio Carlos #6627, ICB-UFMG, Belo Horizonte 31270-010, Minas Gerais, Brazil; 3 Departamento de Bioquímica e Imunologia, Universidade Federal de Minas Gerais, Avenida Antonio Carlos #6627, ICB-UFMG, Belo Horizonte 31270-010, Minas Gerais, Brazil

**Keywords:** *trans*-caryophyllene, rat tracheal smooth muscle, anti-spasmodic, voltage-dependent Ca^2+^ channels

## Abstract

*trans*-Caryophyllene is a major component in the essential oils of various species of medicinal plants used in popular medicine in Brazil. It belongs to the chemical class of the sesquiterpenes and has been the subject of a number of studies. Here, we evaluated the effects of this compound in airway smooth muscle. The biological activities of *trans*-caryophyllene were examined in isolated bath organs to investigate the effect in basal tonus. Electromechanical and pharmacomechanical couplings were evaluated through the responses to K^+^ depolarization and exposure to acetylcholine (ACh), respectively. Isolated cells of rat tracheal smooth muscle were used to investigate *trans*-caryophyllene effects on voltage-dependent Ca^2+^ channels by using the whole-cell voltage-clamp configuration of the patch-clamp technique. *trans*-Caryophyllene showed more efficiency in the blockade of electromechanical excitation-contraction coupling while it has only minor inhibitory effect on pharmacomechanical coupling. Epithelium removal does not modify tracheal smooth muscle response elicited by *trans*-caryophyllene in the pharmacomechanical coupling. Under Ca^2+^-free conditions, pre-exposure to *trans*-caryophyllene did not reduce the contraction induced by ACh in isolated rat tracheal smooth muscle, regardless of the presence of intact epithelium. In the whole-cell configuration, *trans*-caryophyllene (3 mM), inhibited the inward Ba^2+^ current (I_Ba_) to approximately 50% of control levels. Altogether, our results demonstrate that *trans*-caryophyllene has anti-spasmodic activity on rat tracheal smooth muscle which could be explained, at least in part, by the voltage-dependent Ca^2+^ channels blockade.

## 1. Introduction

The terpenes, found in essential oils of different plants, are micro-constituents most commonly used as flavor additives in food, toiletries, and perfumes [1]. Recent studies from different groups around the World have shown that terpenes and terpenoids exert a plethora of pharmacological effects [2]. *trans*-Caryophyllene is an important constituent of the essential oil of several species of plants. It is the major chemical constituent (20.6%) of the essential oil of *Pterodon polygalaeflorus* (EOPp). EOPp blocks the electromechanical excitation-contraction coupling without affecting the pharmacomechanical coupling [3]. The blocking effect caused by EOPp was suggested to result from inhibition of dihidropyridine-sensitive Ca^2+^ channels, but there were no evidence to support that suggestion [3].

*trans*-Caryophyllene has been reported to possess many pharmacological effects. For example, it displays antimicrobial [4] and analgesic activity [5]. It activates the endocannabinoid system [6]. *trans*-Caryophyllene also has a well-documented anti-inflammatory activity [7,8]. Additionally, *trans*-caryophyllene is effective on intestinal smooth muscle, blocking the electromechanical and pharmacomechanical excitation-contraction coupling [9]. Those activities allow it to be considered as a potential anti-spasmodic agent in tracheal smooth muscle.

Not much information is currently available on the potential spasmolytic effects of *trans*-caryophyllene on tracheal smooth muscle. We demonstrate herein that *trans*-caryophyllene has anti-spasmodic effects on rat tracheal smooth muscle. In order to explain this result we hypothesized that *trans*-caryophyllene elicits tracheal smooth muscle relaxation by inhibiting voltage-dependent L-type Ca^2+^ channels.

## 2. Results and Discussion

### 2.1. Effects of trans-Caryophyllene on Basal Tone of Isolated Rat Trachea with or without Epithelium

**Figure 1:**
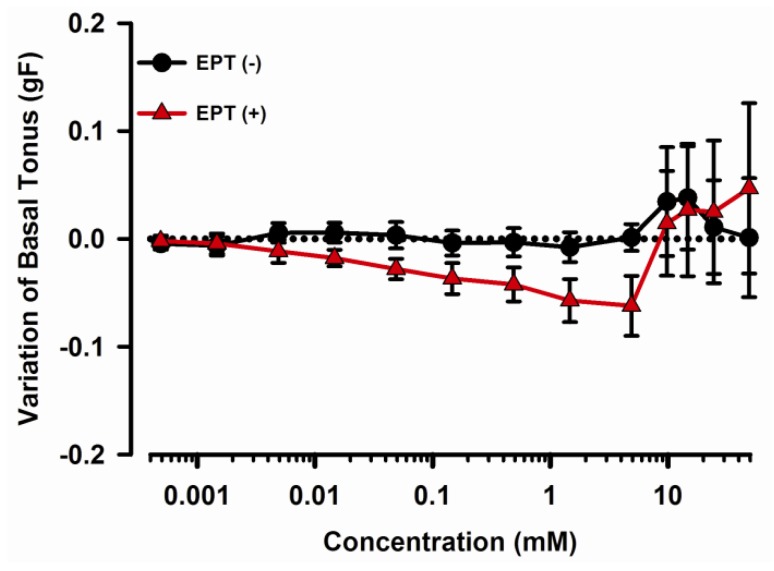
Effects of cumulatively increasing concentrations (0.0005–50 mM) of *trans*-caryophyllene on basal tonus of isolated rat tracheal preparations with (closed triangles; n = 6) or without (closed circles; n = 8) intact epithelium. Ordinate represents the force variation of basal tone (positive deflections means contraction and negative deflections means relaxation). EPT (+) and EPT (−) are preparations without and with epithelium removal, respectively. Symbols indicate means ± S.E.M.

*trans*-Caryophyllene activity on the basal tone of rat tracheal segments with or without epithelium differed. In rat tracheal segments with intact epithelium, *trans*-caryophyllene at the concentration range from 0.05 to 5.0 mM provoked a progressive and significant relaxation (Figure 1; *p* < 0.05; one way ANOVA; n = 6). Interestingly, when we used concentrations from 10 to 50 mM the tendency to provoke relaxation disappeared and the tonus returned to control levels. After epithelium removal, *trans*-caryophyllene had no significant effect on rat tracheal basal tone (Figure 1; n = 8).

### 2.2. Inhibitory Effects of trans-Caryophyllene on Contractions Induced by High-K^+^ in Isolated Rat Trachea with or without Epithelium

**Figure 2:**
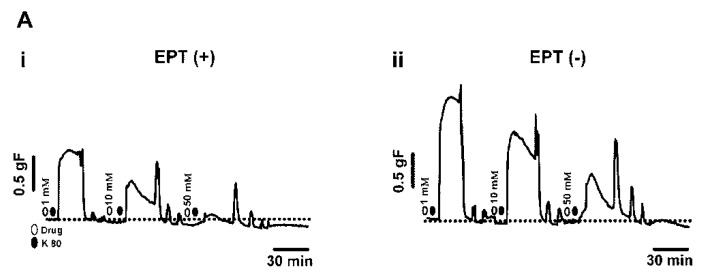

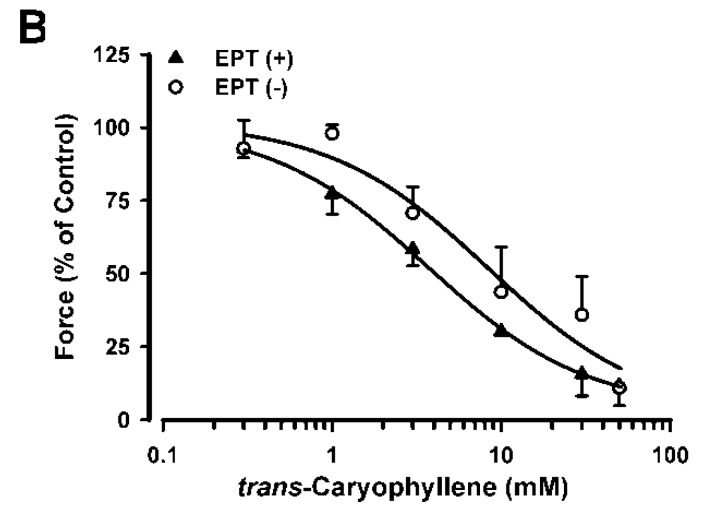
Representative experimental traces showing the relaxant effects of non-cumulatively increasing concentrations of *trans*-caryophyllene (0.3–50 mM) with (EPT(+), **Ai**; n = 5) and without (EPT(−), **Aii**; n = 4) intact epithelium on the high K^+^-induced contraction (K 80) of rat isolated tracheal smooth muscle. (**B**) Concentration-response curves of *trans*-caryophyllene-induced relaxation. Closed triangles represent segments with intact epithelium and open circles without intact epithelium. Symbols are means ± S.E.M.

When non-cumulatively administered to the preparation, *trans*-caryophyllene (0.3 to 50 mM) concentration-dependently and significantly (*p* < 0.05, Two Way ANOVA) decreased the contractions induced by high K^+^ concentration (80 mM) (Figure 2Ai,Aii). 

In the presence of intact epithelium *trans*-caryophyllene showed an IC_50_ value of 3.7 ± 0.4 mM (n = 5). When epithelium was removed the IC_50_ value was 8.4 ± 10.5 mM (n = 4). The results suggest that *trans*-caryophyllene induces relaxation in a concentration-dependent manner (Figure 2B). It seems that the effect is not dependent on the presence of intact epithelium.

### 2.3. Inhibitory Effects of trans-Caryophyllene on Contractions Induced by ACh in Isolated Rat Trachea with or without Epithelium

**Figure 3:**
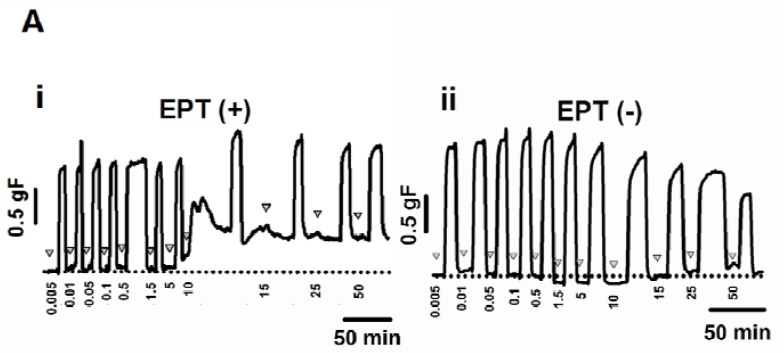

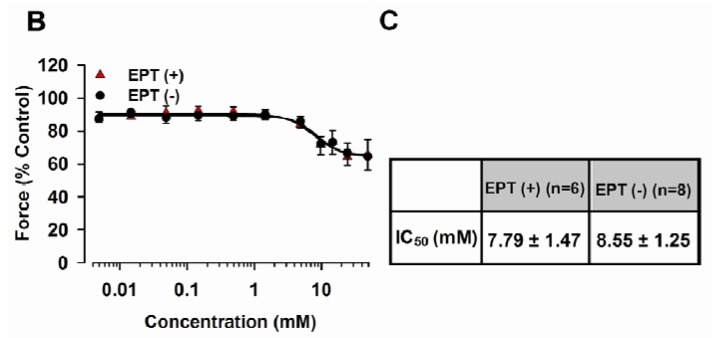
Representative experimental tracings showing the inhibitory effects of non-cumulative increasing concentrations (0.005–50 mM) of *trans*-caryophyllene on the ACh-induced contractions in rat isolated trachea with (EPT(+), **Ai**; n = 6) and without (EPT(−), **Aii**; n = 8) intact epithelium. Inverted triangles indicate drug application. (**B**) Concentration-response curves of the relaxant actions of *trans*-caryophyllene. Closed triangles represent segments with intact epithelium and closed circles without intact epithelium. Symbols are means ± S.E.M. (**C**) Average values for relative IC_50_.

As *trans*-caryophyllene relaxed tracheal smooth muscle (with or without intact epithelium) contracted by 80 mM extracellular K^+^, the possible inhibitory effects of *trans*-caryophyllene on contractions induced by ACh was also investigated. It was found that in tracheal preparations with (Figure 3Ai, n = 6) or without (Figure 3Aii, n = 8) epithelium and pre-exposed for 5 min to a given *trans*-caryophyllene concentration (0.005 to 50 mM), there was inhibition of the contraction induced by sub-maximal concentration of ACh (10 μM) as compared to controls.

This inhibition, however, was only partial, reaching a peak at 30 mM *trans*-caryophyllene, at which concentration the sub-maximal ACh-induced contraction only declined to 64.43 ± 2.78 and 65.03% ± 2.15% of control in preparations with and without epithelium, respectively. The calculated relative IC_50_ values were 7.79 ± 1.47 mM and 8.55 ± 1.25 mM in preparations with and without epithelium, respectively (Figure 3B and 3C).

In segments with (five out of six) or without (six out of eight) epithelium, but previously challenged with ACh (10 µM), application of *trans*-caryophyllene at 10 mM or higher concentrations promoted a sustained contraction. In each of these cases time was allowed to achieve partial subsidizing of *trans*-caryophyllene-induced sustained contraction and for the subsequent contraction induced by ACh. The peak sustained tension was then measured. Peak contraction induced by 10 mM *trans-*caryophyllene in ACh-sensitized preparations reached 0.53 ± 0.06 and 0.31 ± 0.07 gF, with or without intact epithelium, respectively.

### 2.4. Effects of trans-Caryophyllene on Contractions Induced by ACh in Isolated Rat Trachea with or without Intact Epithelium in Ca^2+^-free Solution.

**Figure 4:**
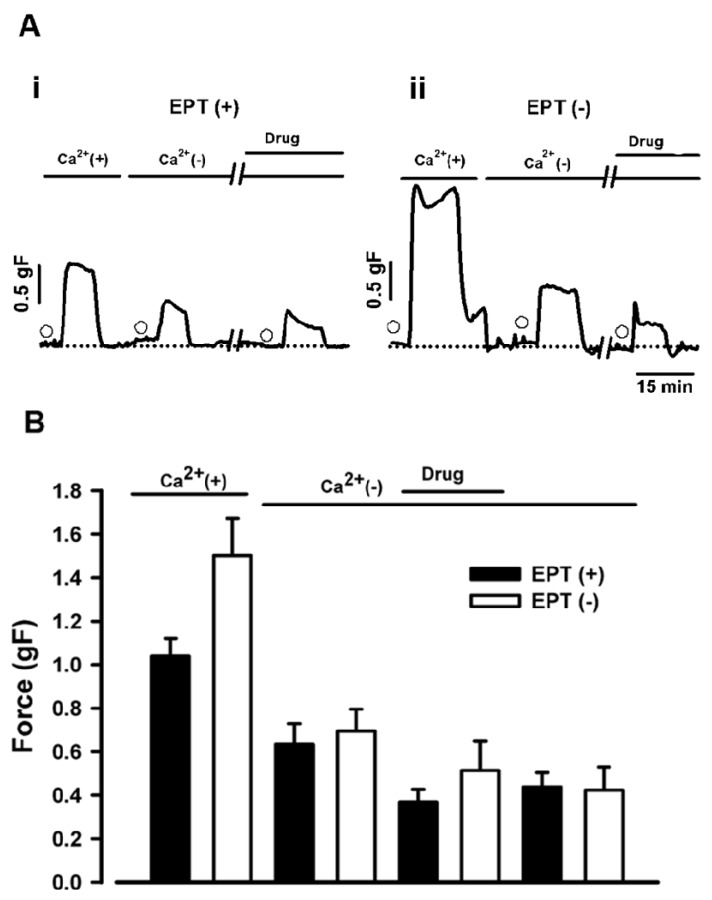
Effect of *trans*-caryophyllene on contractions induced by ACh on preparations maintained in Ca^2+^-free solution. (**A**) Examples of experimental traces obtained from segments where the epithelium is present (EPT(+), **Ai**) or absent (EPT(−), **Aii**). Open circles indicate ACh application at 60 µM. (**B**) Bar graphs showing average of contraction force. Black bars represent mean ± S.E.M. (n = 8) for segments containing intact epithelium. Open bars represent mean ± S.E.M. (n = 8) for segments without intact epithelium.

The effects of *trans*-caryophyllene (10 mM) on ACh (60 μM)-induced contractions in preparations maintained in Ca^2+^-free solution [modified Tyrode’s solution from which Ca^2+^ was removed and EGTA (0.2 mM) was added] were investigated. In segments where epithelial layer was present *trans*-caryophyllene diminished the ACh-induced contraction (Figure 4B, *p* < 0.05). When contractions with (n = 8) or without (n = 8) intact epithelium were compared between themselves and with their respective controls no statistically significant difference (*p* > 0.05, One Way ANOVA) was observed (Figure 4 and Discussion). To avoid misinterpretations due to a possible decrease in ACh contractile response we decided to compare the effects of *trans*-caryophyllene with a second ACh application in Ca^2+^ free conditions (Figure 4, last two bar graphs). Figure 4B shows that *trans*-caryophyllene, under our experimental conditions, did not inhibit pharmacomechanical coupling.

### 2.5. Effects of trans-Caryophyllene on Isolated Tracheal Smooth Muscle Cells

Having established that the *trans*-caryophyllene effect was much more significant on the electromechanical coupling in isolated tracheal smooth muscle preparations, possibly by blocking Ca^2+^ influx, we next investigated whether 3 mM *trans*-caryophyllene [a concentration that blocks approximately 50% of K^+^(80 mM)-induced contraction] could block Ca^2+^ influx by inhibiting L-type Ca^2+^ channels in single rat tracheal smooth muscle cells. To provide direct evidence we performed whole-cell patch-clamp in myocytes to test the effects of *trans*-caryophyllene on L-type Ca^2+^ channels.

**Figure 5:**
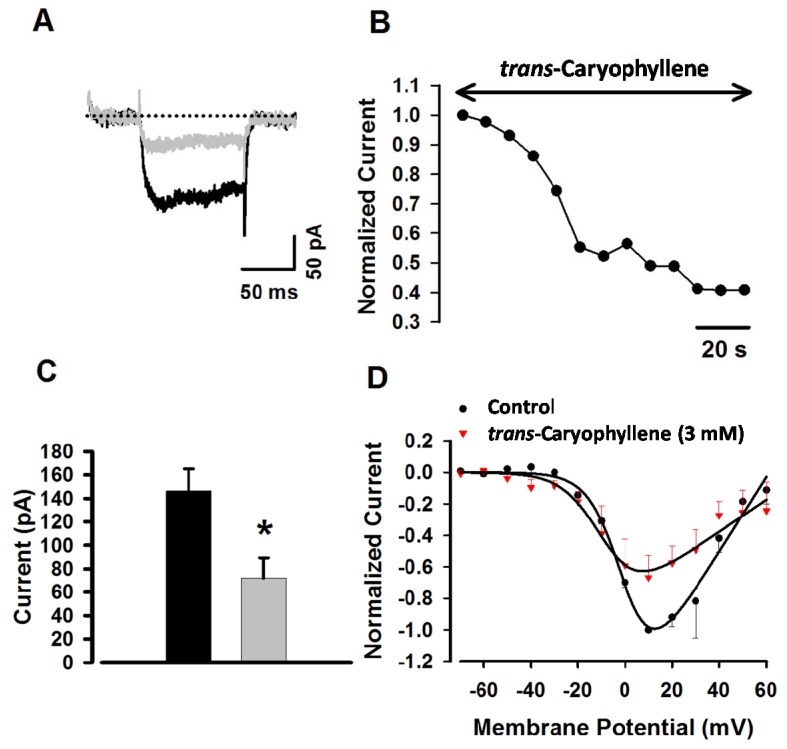
*trans*-Caryophyllene inhibited I_Ba_ in tracheal smooth muscle cells. (**A**) Effect of *trans*-caryophyllene (3 mM) on the I_Ba_ evoked by a depolarizing test pulse to 10 mV from a holding potential of −70 mV. The dotted line denotes zero current. (**B**) Time-course of I_Ba_ blockade caused by *trans*-caryophyllene. Data were normalized to maximum peak current. (**C**) Average I_Ba_ at control (black bar) and after addition of 3 mM *trans*-caryophyllene (gray bar). Bars represent means ± S.E.M, * *p* < 0.05. (**D**) Current-voltage relationships of I_Ba_ in the presence (closed inverted triangles) and absence (closed circles) of trans-caryophyllene. Error bars represent S.E.M.

Figure 5A shows representative examples recorded at 10 mV test potential before (control condition, black trace) and after the application of *trans*-caryophyllene (gray trace). I_Ca,L_ (L-type calcium current) was reduced by approximately 50% of maximal current. To verify the time-course for inhibition provoked by *trans*-caryophyllene, I_Ca,L_ amplitudes were plotted as function of time (Figure 5B). These results were similar to all cells tested (n = 6) and pooled data are depicted in Figure 5C. The current-voltage relationships (I–V) were constructed by applying 100 ms step depolarizations from −70 to +60 mV in 10 mV increments. Figure 5D shows the average I-V in the absence (black symbols, n = 4) and presence of *trans*-caryophyllene (red symbols, 3 mM, n = 4).

### 2.6. Discussion

The current understanding of excitation-contraction coupling in smooth muscle cells indicates that the following two mechanisms may be involved: (1) Those dependent on changes in membrane potential (electromechanical coupling) and (2) those independent of the changes in membrane potential (pharmacomechanical coupling) [10]. Through these mechanisms intracellular Ca^2+^ ([Ca^2+^]_i_ increases and triggers contractions. Thus, [Ca^2+^]_i_ regulates smooth muscle contractions as a result of electro- and pharmacomechanical couplings. To clarify the mechanism of the *trans*-caryophyllene-induced relaxation of airway smooth muscle, we determined the effect of *trans*-caryophyllene on electromechanical and pharmacomechanical coupling. Our study shows for the first time a relaxant effect of *trans*-caryophyllene on airway basal tone in rat isolated trachea. This relaxation of basal tone is dependent of epithelial layer into the tracheal wall. In addition, pre-incubation of trans-caryophyllene caused a concentration-dependent decrease in high K^+^ depolarization-induced and ACh-induced contractions. Conversely, epithelium removal did not change the relaxation provoked by *trans*-caryophyllene. Overall, these findings corroborate our previous studies exploring the effects on *trachealis* muscle of an essential oil having *trans*-caryophyllene as its major constituent [3].

It is generally known that prostanoids synthesized by epithelial cyclo-oxygenases (COX) play a major role in the control of airway contractility since COX inhibitors diminish the airway basal tone [11,12,13,14,15,16,17]. *trans*-Caryophyllene decreased the basal tone in rat *trachealis* muscle only when epithelial layer was preserved. This observation could be explained by a direct inhibitory effect of *trans*-caryophyllene on the COX or by causing an imbalance between relaxant and constrictor prostanoids synthesized by epithelial COX. The absence of *trans*-caryophyllene-induced relaxant activity on preparations without intact epithelium is consistent with these assumptions. Further studies conducted with selective antagonists are needed to clarify this point.

Membrane depolarization by high extracellular K^+^ concentrations induces contraction of airway smooth muscle that is easily relaxed by Ca^2+^ channel antagonists [18] and largely abolished in a Ca^2+^ free medium [18] indicating that high K^+^-elicited contraction is triggered by Ca^2+^ influx through activation of voltage-operated Ca^2+^ channels. We showed that pre-incubation of *trans*-caryophyllene caused a concentration-dependent decrease in high K^+^ elicited contractions suggesting that a blockade of voltage-operated Ca^2+^ channels could be involved. We would argue that *trans*-caryophyllene may have exerted its effect by directly acting on airway smooth muscle cells. This idea is rather likely because mechanical removal of the epithelium did not alter the effect of *trans*-caryophyllene. Therefore, the inhibition of airway smooth muscle contraction elicited by high K^+^ may not requires epithelium-derived relaxing factors.

In the current study, the application of ACh on rat tracheal strips increased tension. This response is a composite of two phases, a rapid raise (which is assumed to be dependent on Ca^2+^ influx and Ca^2+^ release from internal stores) followed by a sustained component (produced mainly by the influx of extracellular Ca^2+^) [19]. In the presence of extracellular Ca^2+^, pre-incubation of *trans*-caryophyllene promoted only a minor inhibition (about 20% at concentrations above 10 mM) of ACh-induced contractions in preparations, with or without intact epithelium. We examined *trans*-caryophyllene effects on Ca^2+^ release elicited by exposure to ACh in the absence of extracellular Ca^2+^. It is worth noting that ACh causes Ca^2+^ release by pharmacomechanical coupling mediated by inositol 1,4,5-trisphosphate [16,20]. In the absence of extracellular Ca^2+^, *trans*-caryophyllene was not found to have any inhibiting effect on contractions induced by ACh. These results indicate that *trans*-caryophyllene do not affect Ca^2+^ release from intracellular stores.

The lack of effect on ACh-induced contractions is consistent with the hypothesis of *trans*-caryophyllene activity being largely restricted to voltage-dependent Ca^2+^ channels, since only a minor part of ACh-induced contraction is due to depolarization and Ca^2+^ influx through these channels [13]. In order to evaluate this hypothesis we used the patch-clamp technique in dissociated cells of the *trachealis* muscle to make a direct measurement of *trans*-caryophyllene effect on voltage-dependent Ca^2+^ currents. Ba^2+^ was used because this ion permeates the voltage-dependent Ca^2+^ channels but not the receptor-operated Ca^2+^ channels and it blocks K^+^ channels [14,15]. These experiments (Figure 5) showed that *trans*-caryophyllene, in a concentration in which it decreases high K^+^-induced contractions, also blocked the voltage-dependent Ca^2+^ current.

This profile presented by *trans*-caryophyllene, which shows a predominant activity on voltage-dependent Ca^2+^ channels, has not been found in other biological preparations. For example, we did find evidences for a relative selectivity of *trans*-caryophyllene activity on voltage-dependent Ca^2+^ channels in ileum [9]. *trans*-Caryophyllene is a major component in many essential oils from plants that are reported to be used to treat inflammation and asthma [21]. Our study is important because it shows, by the first time, the antispasmodic effect elicited by *trans*-caryophyllene in the airway smooth muscle due to voltage-dependent Ca^2+^ channels blockage. 

Recently, clinicians have been seeking additional options other than the currently available conventional treatments to improve the condition of patients with asthma and to spare systemic corticosteroid administration. Therefore, new drugs such as calcium channel inhibitors have been developed to treat asthma-related symptoms [22]. We think that our study could raise the possibility of *trans*-caryophyllene be used as a lead compound for this purpose, especially because it has very low toxicity.

## 3. Experimental 

### 3.1. Animals

Wistar male rats (*Rattus novergicus*), weighing 250–350 g were used. Animals were bred and housed in the animal facility of State University of Ceará under a controlled environment (23–25 °C). Experiments performed were in accordance with the Conselho Nacional de Controle de Experimentação Animal (CONCEA) and US guidelines (NIH publication #85-23, revised in 1996) and received previous approval by local ethical committee on use of animals for experimentation (Comitê de Ética para o Uso de Animais em Pesquisa CEUA/UECE – Protocol # 06379067-0). 

### 3.2. Drugs

Acetylcholine (ACh) was used at 10 and 60 μM to elicit contractions in 2.0 and in 0.0 mM calcium concentration, respectively. High extracellular K^+^ concentration (80 mM) was used to elicit contractions. The inhibitory effects of *trans*-caryophyllene were investigated by pre-incubating it in the tissue bath chamber. Bath final *trans*-caryophyllene concentrations were prepared by dilution of 245 mM *trans*-caryophyllene stock solution. This stock solution was prepared by diluting *trans*-caryophyllene directly in Tween 80 (dispersant agent) and afterwards in Tyrode’s solution [composition in mM: NaCl 136.0, KCl 5.0, MgCl_2_ 0.98, CaCl_2_ 2.0, NaH_2_PO_4_ 0.36, NaHCO_3_ 11.9 and glucose 5.5 (pH 7.4)]. The equivalent Tween 80 concentration had a very small effect on pre-contracted tracheal strips (<5%, data not shown).

Drugs [ACh, *trans*-caryophyllene, ethylene glycol-bis(aminoethylether)-*N*,*N*,*N*',*N*'-tetraacetic acid (EGTA), *N*-2-hydroxyethylpiperazine-*N'-*2-ethanesulfonic acid (HEPES), tetraethylammonium chloride (TEA), adenosine 5'-triphosphate disodium (Na_2_-ATP), papain, bovine serum albumin (BSA), dithiothreithol (DTT), collagenase type II and hyaluronidase] were purchased from Sigma Chemical Company (St. Louis, MO, USA) and from Reagen (Colombo, Paraná, Brazil). All drugs and salts were of analytical grade.

### 3.3. Isolated Tissue Preparation

The animals were sacrificed by cervical dislocation. To study the tracheal muscle relaxation tracheal ring strips were then mounted in a 5 mL tissue bath containing modified Tyrode’s solution maintained at 37 °C. Under baseline tension of 1.0–1.15 gF the isolated tissues were allowed to equilibrate for 1–2 h before the addition of any substance. Experiments were performed on isolated tracheal smooth muscle preparations with or without intact epithelium. Isometric contractions were recorded through a force displacement transducer (Grass, FT-03, Quincy, MA, USA) coupled to an integrated organ bath system attached to a computer (WinDaq, version 1.65, DATAQ Instruments, Akron, OH, USA).

#### 3.3.1. Effects of *trans*-Caryophyllene on Tracheal Basal Tone

In order to assess the effect of the *trans*-caryophyllene on tracheal basal tone, isolated tracheal rings with or without intact epithelium were exposed to increasing concentrations of *trans*-caryophyllene (0.0005–50 mM). The tracheal epithelium was mechanically removed by rubbing with a cotton bud and it was confirmed by measurement of contraction’s magnitude promoted by KCl 60 mM. Contractions above 1300 gF were considered without functional epithelium.

#### 3.3.2. Effects of *trans*-Caryophyllene on the Contractions Induced by ACh and KCl in Isolated Trachea Maintained in Ca^2+^-Containing Medium

In this series of experiments, effects of non-cumulative increasing concentrations of *trans*-caryophyllene (0.005–50 mM) on the contractile responses to ACh (10 μM) and (0.3–50 mM) to K^+^ (80 mM) were studied in rat tracheal smooth muscle maintained in Ca^2+^-containing medium.

#### 3.3.3. Effects of *trans*-Caryophyllene on ACh-Induced Contraction of Rat Isolated Trachea Maintained in Ca^2+^-Free Medium

Ca^2+^ availability from extracellular Ca^2+^ entry was indirectly evaluated by recording the contraction induced by ACh in Ca^2+^-free medium (containing 0.2 mM EGTA). After the equilibration period, ACh at 60 μM (a concentration able to promote contractions in Ca^2+^ free medium with amplitude large enough to allow good resolution) was added to the bath and produced a contraction that was established as control response. After washing preparation again with calcium free solution, *trans*-caryophyllene (10 mM) was added to the preparations. Afterwards, in the presence of *trans*-caryophyllene the preparation was challenged with ACh (60 μM). As there is a possibility of time-dependent decrease in the ACh elicited smooth muscle contraction we compared *trans*-caryophyllene effects with the response to a second application of ACh. In all experimental series external controls (tracheal ring strips exposed only to vehicle) were done.

### 3.4. Isolation of Single Tracheal Smooth Muscle Cells

A 15-cm segment of trachea was immediately removed and placed at room temperature in a low-Ca^2+^ physiological salt solution (PSS) in mM: 137 NaCl, 5.6 KCl, 0.44 NaH_2_PO_4_, 0.42 Na_2_HPO_4_, 4.17 NaHCO_3_, 3.55 MgCl_2_, 0.05 CaCl_2_, 10 HEPES and 5 glucose. The tracheal muscle was dissected away from the cartilage and minced with scalpel blades. Tracheal smooth muscle strips were divided and transferred to 3 mL of dissociation solution containing normal PSS (in mM): 137 NaCl, 5.6 KCl, 0.44 NaH_2_PO_4_, 0.42 Na_2_HPO_4_, 4.17 NaHCO_3_, 1 MgCl_2_, 2.6 CaCl_2_, 10 Hepes and 5 glucose, papain (0.9 mg/mL), BSA (1 mg/mL), and DTT (0.9 mg/mL). The strips were placed in a 37 °C water bath during 60 min and minced every seven-minute interval. After, tracheal smooth muscle strips were transferred to 3 mL of low-Ca^2+^ PSS supplemented with collagenase (1 mg/mL, type II), BSA (1 mg/mL) and hyaluronidase (0.9 mg/mL) for further digestion. The strips were placed in a 37 °C water bath during approximately 30 min and minced every seven-minute interval. All enzymes were obtained from Sigma Chemical Co. The solution was centrifuged at 1,500 revolutions/min for 1 min three times and washed twice with low-Ca^2+^ PSS. Afterwards, cells were resuspended in normal PSS and triturated with a pipette to release individual smooth muscle cells from the tissue. The solution containing the dissociated cells was poured onto glass coverslips, and the cells were allowed to adhere for 40–60 min at room temperature.

### 3.5. Membrane Current Recordings

The standard bath solution employed in the whole-cell voltage-clamp experiments contained the following composition (in mM): 130 NaCl, 20 BaCl_2_, 0.5 MgCl_2_, 10 HEPES and 5 glucose (pH 7.4 corrected with NaOH). Currents were compared in the presence and absence of *trans*-caryophyllene (3 mM). Pipettes were pulled from glass capillaries (Perfecta, São Paulo, SP, Brazil) with a micropipette puller (PP-830, Narishige, Tokyo, Japan). They had resistances of 2–4 MΩ (average 3.4 ± 0.3 MΩ) when filled with pipette solution (in mM: 130 CsCl, 10 TEA, 10 EGTA, 4 MgCl_2_, 10 Hepes and 4 Na_2_ATP). An Ag–AgCl wire was used as reference electrode.

An EPC-9 patch clamp amplifier (HEKA Instruments, Lambrecht/Pfalz, Germany) and pulse software were used to record whole-cell Ca^2+^ channel currents. Capacitive currents were compensated electronically and a P/4 protocol was used for linear leak and residual capacitance subtraction. Ca^2+^ currents were low-pass filtered at 3 kHz and sampled at 10 kHz. Patch clamp experiments were performed on 35-mm Petri dishes using an inverted microscope (Axiovert 20, Carl Zeiss, Göttingen, Germany). The bath was continuously perfused at 1–2 mL/min throughout the experiment. Solutions were gravity fed to the input ports of a solenoid valve mounted close to the bath, which was used to choose between one of two solutions. 

Currents through high-voltage-activated (HVA) Ca^2+^ channels were evaluated using Ba^2+^ (*I*_Ba_) as the charge carrier. The currents were generated by 100 ms steps to 10 mV from a holding potential of −80 mV. To determine current–voltage relationships, cells were held at holding potential of −80 mV and subjected to step depolarizations of 100 ms from −70 to +60 mV in 10 mV increments every 10 s. Data were collected after the whole-cell configuration was obtained and current amplitude had stabilized, usually approximately 3 min after rupture of the cell membrane. Only cells with an input resistance >1 GΩ with no substantial rundown were analyzed. 

### 3.6. Statistical Analysis

All values were presented as mean ± S.E.M., and statistical comparisons were made using the paired Student’s *t* test, one-way and two-way analysis of variance (ANOVA). *p* < 0.05 was considered to be significant.

## 4. Conclusions

In conclusion, we have clearly demonstrated that *trans*-caryophyllene has anti-spasmodic activity on *trachealis* muscle. We also demonstrated by means of direct electrophysiological measurements (using the patch-clamp method) that *trans*-caryophyllene-induced blockade of Ca^2+^ influx through voltage-dependent Ca^2+^ channels. The provided information that will certainly enrich the pharmacological profile of *trans*-caryophyllene, a substance that thus appears as a candidate worthy of more investigations envisaging its pharmacotherapeutic use.
